# ReQON: a Bioconductor package for recalibrating quality scores from next-generation sequencing data

**DOI:** 10.1186/1471-2105-13-221

**Published:** 2012-09-04

**Authors:** Christopher R Cabanski, Keary Cavin, Chris Bizon, Matthew D Wilkerson, Joel S Parker, Kirk C Wilhelmsen, Charles M Perou, JS Marron, D Neil Hayes

**Affiliations:** 1 Department of Statistics and Operations Research, Chapel Hill, NC, USA; 2 Renaissance Computing Center, Chapel Hill, NC, USA; 3 Lineberger Comprehensive Cancer Center, Chapel Hill, NC, USA; 4 Department of Genetics, Chapel Hill, NC, USA; 5 Department of Internal Medicine, Division of Medical Oncology, Multidisciplinary Thoracic Oncology Program, University of North Carolina at Chapel Hill, Chapel Hill, NC, USA

**Keywords:** Next-generation sequencing, Quality score, Recalibration, Bioinformatics, Bioconductor

## Abstract

**Background:**

Next-generation sequencing technologies have become important tools for genome-wide studies. However, the quality scores that are assigned to each base have been shown to be inaccurate. If the quality scores are used in downstream analyses, these inaccuracies can have a significant impact on the results.

**Results:**

Here we present ReQON, a tool that recalibrates the base quality scores from an input BAM file of aligned sequencing data using logistic regression. ReQON also generates diagnostic plots showing the effectiveness of the recalibration. We show that ReQON produces quality scores that are both more accurate, in the sense that they more closely correspond to the probability of a sequencing error, and do a better job of discriminating between sequencing errors and non-errors than the original quality scores. We also compare ReQON to other available recalibration tools and show that ReQON is less biased and performs favorably in terms of quality score accuracy.

**Conclusion:**

ReQON is an open source software package, written in R and available through Bioconductor, for recalibrating base quality scores for next-generation sequencing data. ReQON produces a new BAM file with more accurate quality scores, which can improve the results of downstream analysis, and produces several diagnostic plots showing the effectiveness of the recalibration.

## Background

**Figure 1:**
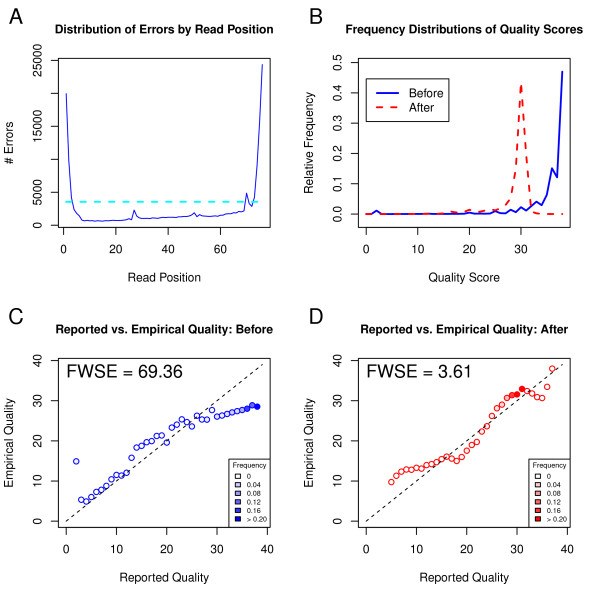
**Recalibration of U87 cell line replicate 1 with ReQON.** Plot **A** shows the distribution of sequencing errors by read position. Plot **B** shows frequency distributions of quality scores before (solid blue) and after (dashed red) recalibration. Reported quality scores versus empirical quality scores are shown before (plot **C**) and after (plot **D**) recalibration. The points are shaded according to the frequency of bases assigned that quality score, corresponding to the values shown in plot **B**. Plots **C** and **D** also report the Frequency-Weighted Squared Error (FWSE), a measure of quality score accuracy. The large decrease in FWSE confirms that the recalibrated quality scores more accurately represent the probability of a sequencing error than the original quality scores.

Next-generation sequencing (NGS) technologies are important tools for studying genome-wide DNA and RNA expression, Single Nucleotide Polymorphisms (SNPs), mutations and alternative splicing [1]. When a sequencer calls a specific base, there is a small chance that it will make an error and call an incorrect base. This sequencing error rate is machine, run and sample specific, but it occurs at a rate of approximately 1/1000 [2], resulting in tens of millions of errors in a single experiment. A quality score is also provided for each base, corresponding to the probability of a sequencing error. Unfortunately, it has been shown that the reported quality scores are inaccurate ([3,4] and our Figure 1C). Thus, it is essential to recalibrate base quality scores so that they more accurately reflect the probability of a sequencing error. Incorporating these recalibrated quality scores into downstream analyses, such as variant calling, can produce more accurate and confident results [5]. 

Ewing and Green [6] propose a method, Phred, which calibrates quality scores using fluorescence intensity data. However, many labs do not have the resources to store fluorescence intensity data and are unable to use such calibration tools. Therefore, there is a need to recalibrate the quality scores that are produced by the sequencer machine. There exist recalibration tools that only require aligned sequence data, with two of the most popular being GATK [3] and the BAQ option in SAMtools [7], which both run on Unix.

Here we present a novel R package, ReQON (Recalibrating Quality Of Nucleotides), that recalibrates the quality scores from a BAM file of aligned sequencing data. ReQON also produces diagnostic plots (Figure 1) that identify read positions with a significant number of errors, visualize the distribution of quality scores before and after recalibration, and show the improvement in accuracy of the recalibrated quality scores. ReQON uses a different model than other available recalibration tools and proposes an improved statistic for comparing recalibration performance. To our knowledge, ReQON is the first recalibration tool written in R.

## Implementation

ReQON is open source software, written in R and available through the Bioconductor project [8]. This section describes the ReQON algorithm developed to recalibrate base quality scores.

### Input

The input to ReQON is an indexed and sorted BAM file [9] with quality scores reported in the QUAL field. The reads can be aligned to any genome using any alignment algorithm. The recalibration results will be dependent on the accuracy of these alignments.

### Algorithm

ReQON uses logistic regression to recalibrate the quality scores. The following parameters must be specified:

· Region: Genomic coordinates of the region that the regression model is trained on. This training region must be large enough to obtain accurate coefficients for the regression model. On the other hand, specifying a larger than necessary region will increase run time and may overfit the model to the training set. We recommend training on one of the smaller chromosomes, or specifying MaxTrain, described next.

· MaxTrain (optional): This allows the user to train on a fixed number of bases from a large training region. For example, training on the first 10 million bases of the genomic region given in Region is achieved by setting MaxTrain = 10,000,000. Typically, results are consistent when training on at least 10 million bases and do not improve when the training size is larger than 25 million bases.

· RefSeq: File containing the reference sequence corresponding to the training set.

· SNP (optional): File of known variant locations to remove from the training set before recalibration.

· nerr: The maximum number of errors tolerated at a genomic position (default = 2). Positions with more than nerr errors may likely be true variants, so bases from these positions are removed from the training region.

· nraf: The maximum non-reference allele frequency at a genomic position that is allowed (default = 0.05). Positions with non-reference allele frequency greater than nraf are removed from the training set for the same reason as nerr.

The first step is to read the training region, specified by Region and MaxTrain, from the input BAM file. Positions that are listed in SNP are removed from the training set. Next, sequencing errors are identified as bases that do not match RefSeq. In reality, some of these identified bases will not be errors but instead correct calls, such as novel variants or mapping errors. In an attempt to remedy this issue, two different thresholds are set to remove positions most likely to contain false error calls. At each genomic position, thresh = max{nerr, nraf × coverage} is calculated. This threshold sets a maximum number of allowable called errors for positions with low coverage, and a maximum frequency of non-reference bases for positions with high coverage. The default settings allow up to 2 errors per position if the coverage is less than 60x, and more errors (0.05 × coverage) for positions with at least 60x coverage. Positions with more called errors than thresh are identified and removed from the training set. Note that when we say that “a position is removed from the training set,” this is different than keeping the bases at this position in the training set but switching the call from error to non-error. Instead, all bases at this position are removed from the training set due to low confidence of the error call.

Every base in the training set has now been classified as either error or non-error. There is a strong relationship between errors and their position in the read. Most errors occur at the end of the read, due to technical aspects of the sequencing process [10]. This same pattern is present in Figure 1A, which shows the distribution of sequencing errors in the training set by their read position. The rest of Figure 1 is described in more detail in the next section. To account for these high-error read positions in the ReQON regression model, the algorithm flags read positions that have more errors than a specified threshold, set at 1.5 times the average number of errors per read position. This threshold is visualized by the dashed cyan line in Figure 1A. For notational purposes, assume that there are *k* flagged positions, denoted as *fp*_*1*_*fp*_*2*_, … *fp*_*k*_.

Regression coefficients (βs) are obtained from the following logistic regression model,$logit\left(\mathrm{Probability}\;\mathrm{of}\;Y=1\right)=\beta_{0}+\beta_{1}X_{1}+\beta_{2}X_{2}+\beta_{3}X_{3}+\beta_{4}X_{4}+\beta_{5}X_{5}+\beta_{6}X_{6}+\beta_{7}X_{7}+\beta_{8}X_{8}+\cdot \cdot \cdot +\beta_{7+k}X_{7+k}$,

where Y is the (*n* x 1) response vector and the *X*s are (*n* x 1) vectors of explanatory variables, as defined below.

· *Y*(*i*) = I{base *i* is a sequencing error (i.e., does not match RefSeq)}.

· *X*_1_(*i*) = original quality score of base *i.*

· *X*_2_(*i*) = I{*X*_1_(*i*) = 0}. Many base calling algorithms assign a quality of zero to indicate bad or randomly called bases, so these bases are treated separately.

· *X*_3_(*i*) = average quality score across all bases in the read containing base *i*. This helps identify reads for which all bases are assigned low quality scores.

· *X*_4_(*i*) = Read position of base *i*.

· *X*_5_(*i*) = I{base *i* = A}.

· *X*_6_(*i*) = I{base *i* = C}.

· *X*_7_(*i*) = I{base *i* = G}.

· *X*_7+m_(*i*) = I{*X*_4_(*i*) = *fp*_*m*_} for *m* = 1, …, *k*. These indicator variables identify bases originating from one of the flagged read positions.

Additional covariates were originally considered, but those that did not improve model performance were dropped from the final model.

Due to memory constraints, it is not practical to fit the model when the training set contains tens of millions of bases. Instead, the training set is split into smaller subsets of no more than 10 million bases and the regression model is run on all subsets. Then, for each coefficient, the median is calculated over all regression models. Once these median regression coefficients are obtained, the model is applied to each base in the input BAM file to obtain predicted error probability values. These probabilities are transformed to the Phred scale [6] and rounded down to the nearest integer.

### Output and visualizations

ReQON outputs a BAM file with original quality scores replaced by the recalibrated scores. Additionally, ReQON produces diagnostic plots (Figure 1) which show the effectiveness of the quality score recalibration on the training set. Plot A shows the distribution of errors in the training set by their read position. Any read position above the threshold (dashed cyan line) is given an additional indicator variable in the regression model, discussed in the previous section. Plot B shows the relative frequency distribution of quality scores both before (solid blue line) and after (dashed red line) recalibration.

The bottom two plots show the reported quality score versus the empirical quality score before (plot C) and after (plot D) recalibration. The empirical quality score is calculated by computing the observed error rate for all bases in the training set that are assigned a specific quality score. This error rate is then converted to the log-transformed Phred scale. If the quality scores are accurate, which occurs when the observed and reported sequencing error rates match, the points will fall on the 45-degree line.

The bottom plots also report the Frequency-Weighted Squared Error (FWSE), a measure of how close the points lie to the 45-degree line. FWSE is calculated by squaring the error (vertical distance between the point and 45-degree line), weighting this squared error by its relative frequency (shown in plot B and represented by shading of the point) and summing across all quality scores. FWSE will be close to zero if the quality scores accurately reflect the probability of a sequencing error.

## Results

### Accuracy

As an example, two replicates of RNA from the U87 glioblastoma cell line [11] were sequenced using Illumina’s Genome Analyzer II, representing identical sequence runs but of slightly differing quality. For both cell line replicates, ReQON was run using the default settings with the model trained on chromosome 10.

Figure 1 shows the diagnostic output of ReQON after recalibration. Plot A shows that a majority of the sequencing errors occur at the ends of the read. Plot B shows that the majority of quality scores before recalibration were larger than 35, with almost 50% of the bases receiving a quality score near 40. After recalibration, the quality scores are assigned smaller values, with most quality scores falling between 25 and 35. Plot C confirms that the original quality scores are not very accurate because the quality scores with the largest frequencies (shaded dark blue) are far from the 45-degree line and, thus, FWSE is large. For example, plot B shows that approximately 50% of the bases are assigned a quality score of 40. In plot C, the empirical quality of this reported score is around 30. Thus, this single quality score contributes approximately 0.5(40 – 30)^2^ = 50 to the total FWSE of 69.36. Plot D shows that, after recalibration, the quality scores do a much better job of representing true sequencing error rates, reflected by the 95% decrease in FWSE. Some of the lower quality scores lie away from the 45-degree line, but because few bases are assigned these scores (represented by unshaded circles), they contribute little to FWSE. The high-frequency quality scores (shaded dark red) are very close to the 45-degree line, resulting in a total FWSE that is very small.

Table 1 shows the FWSE values of the training set (chromosome 10) for both cell line replicates before and after recalibration with ReQON. ReQON decreases FWSE by over 90% for both replicates. Because ReQON is trained on a small subset of the genome, model performance can be assessed on another region of the genome not used in training, which we will refer to as a testing set. Table 1 also reports FWSE before and after applying ReQON to an independent testing set (chromosome 20). In each case, FWSE of the recalibrated quality scores is approximately the same for both the training and testing sets. This demonstrates that ReQON does not overfit the model to the training set and that recalibrated quality scores accurately represent the probability of a sequencing error.

**Table 1:** Comparison of Frequency-Weighted Squared Error (FWSE)

		**Original**	**ReQON**	**GATK**	**BAQ**
Chromosome 10	Replicate 1	69.36	3.61	11.55	13.93
	Replicate 2	62.89	5.31	14.91	21.76
Chromosome 20	Replicate 1	71.09	3.04	12.28	15.93
	Replicate 2	64.34	5.82	17.43	24.38

Comparison of FWSE for two cell line replicates between original quality scores reported from the sequencer machine and after recalibration with ReQON, GATK and BAQ. ReQON quality scores have the lowest FWSE values, corresponding to increased accuracy. ReQON does not overfit the model to the training set, shown by the roughly equivalent FWSE values for both the training (chromosome 10) and testing (chromosome 20) sets after recalibration.

### Discrimination

Another desirable property of quality scores is the ability to separate sequencing errors from true variants. To perform this analysis, all bases that do not match RefSeq (hg 19) are analyzed. These non-reference bases are further classified as true variants (reported in dbSNP version 132 [12]) or sequencing errors (not in dbSNP132). Similar to the training set, bases identified as sequencing errors are removed if there are more than two errors at a position (or allele frequency greater than 0.05 for high coverage positions) as these may represent novel variants or systematic alignment errors. To increase confidence in the true variant calls, positions identified as true variants with less than 3 non-reference bases (or allele frequency less than 0.05 for high coverage positions) are also removed, as these may actually be sequencing errors.

**Figure 2:**
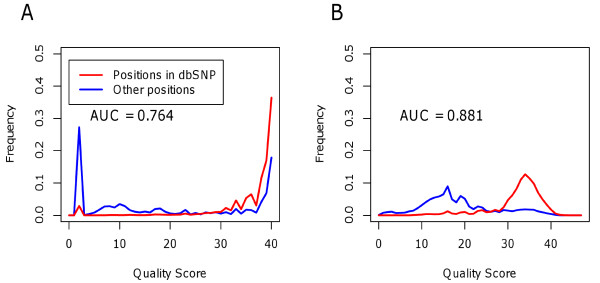
**Discrimination performance of original and ReQON-recalibrated quality scores.** Relative frequency distributions of quality scores for bases not matching the reference sequence in chromosome 20 of cell line replicate 2. These non-reference bases are separated as belonging to positions in dbSNP version 132 (known variants, red curve) versus other positions (sequencing errors, blue curve). Plot **A** shows the distribution of original quality scores and plot **B** shows the distribution after recalibration with ReQON. The area under the ROC curve (AUC) is reported. The increased AUC demonstrates that the recalibrated quality scores do a better job of distinguishing sequencing errors from non-errors.

Classification performance is measured by the area under the corresponding receiver operating characteristic (ROC) curve, or AUC. Figure 2 plots the relative frequency distributions of quality scores for non-reference bases in chromosome 20, an independent testing set. The red curve plots the distribution of bases belonging to positions in dbSNP (13,491 bases) and the blue curve plots the distribution of bases identified as sequencing errors (83,180 bases). Plot A shows the distribution of original quality scores before recalibration. The AUC for the original quality scores is 0.764, representing reasonable separation between the two classes. Plot B shows the distribution of quality scores after recalibration with ReQON. The two curves now have very different distributions. The quality scores of most bases at positions in dbSNP (red) have high quality scores (above 25). It is possible that many of the lower quality bases at positions in dbSNP may be sequencing errors, as bases belonging to any third alleles were not filtered out. In contrast, the quality scores of sequencing errors (blue) are mostly below 25. This better separation between the two classes is supported by the increased AUC (0.881 vs. 0.764). This analysis shows that, in addition to providing quality scores that more accurately reflect the probability of a sequencing error, the recalibrated quality scores also do a better job of distinguishing sequencing errors from true variants. The AUC statistics for both cell line replicates are shown in Table 2.

**Table 2:** Comparison of the area under the ROC curve (AUC)

	**Original**	**ReQON**	**GATK**	**BAQ**
Replicate 1	0.673	0.806	0.824	0.798
Replicate 2	0.764	0.881	0.874	0.814

Comparison of AUC for two cell line replicates recalibrated with ReQON, GATK and BAQ. Bases from chromosome 20 that do not match the reference sequence are separated as belonging to positions in dbSNP version 132 or not. Overall, all three recalibration methods outperform the original quality scores. ReQON and GATK have similar AUC values, with both methods outperforming BAQ.

### Comparison to GATK and BAQ

There are other available computational tools that recalibrate base quality scores. For example, some variant calling tools, such as Atlas-SNP2 [13] and SOAPsnp [4], recalibrate the quality scores as part of their algorithm. These tools incorporate the recalibrated qualities into their method, but do not output a file with the recalibrated scores. Therefore, we are not able to compare their performance to ReQON.

The most popular recalibration tool is embedded in the Genome Analysis Toolkit (GATK) [3]. There are four main differences between the recalibration algorithms of ReQON and GATK.

1. GATK runs on Unix. ReQON is an R package.

2. GATK does not recalibrate bases originally assigned a low quality score, with the default threshold set at 5. ReQON recalibrates all bases regardless of original quality score.

3. GATK requires a reference sequence file for the entire genome and trains its model on all bases in the input BAM file. ReQON allows the user to specify a smaller region of the genome (such as a chromosome) to train the model, only requiring reference sequence for this training region.

4. GATK identifies sequencing errors by filtering out known variant positions, then calling all bases that do not match the reference sequence as errors. While reasonable on average, some of these non-reference bases will not be sequencing errors but instead novel variants or systematic mapping errors. In contrast, ReQON utilizes information from multiple reads by removing positions from the training set that do not pass quality thresholds (determined by nerr and nraf).

**Figure 3:**
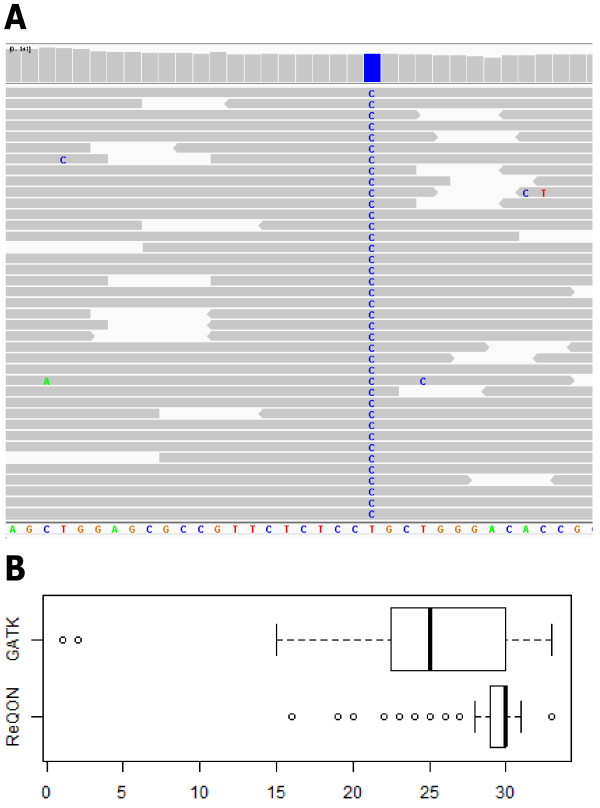
**Example position where bases are identified as sequencing errors by GATK but not ReQON.** Plot **A** shows an Integrative Genomics Viewer (IGV) visualization of chr10:75,531,679-75,531,712 for cell line replicate 1, highlighting a position where the reference sequence is T but all of the bases mapped to this position are a **C**. This position (chr10:75,531,700) is not listed as a known variant in dbSNP version 132. The bases at this position are removed from the training set by ReQON but are called as sequencing errors by GATK. Plot **B** shows box plots comparing the quality scores of the bases at this position after recalibration with GATK and ReQON. Overall, ReQON assigns higher quality scores to these non-reference bases than GATK.

Figure 3 shows an example of a position in the training set where the bases do not match the reference sequence and the position is not listed in dbSNP version 132. Plot A visualizes the position and surrounding region using the Integrative Genomics Viewer (IGV) [14]. At the position, the reference sequence is a T, but all of the 103 bases mapped to this position are C (colored blue). This position most likely represents a novel variant or systematic mapping error. These bases are removed from the training set by ReQON because this position has a higher non-reference allele frequency (100%) than nraf (set at 5%). However, GATK calls all of these non-reference bases as sequencing errors when training its model. Plot B shows boxplots of the quality scores of the bases at this position after recalibrating with ReQON and GATK. The quality scores assigned by ReQON are significantly higher than the quality scores assigned by GATK (two-sided paired *t*-test, p = 8.36 × 10^-9^). 

Another commonly used recalibration tool is BAQ (Base Alignment Quality) [7]. BAQ uses Hidden Markov Models to accurately measure the probability of a base being incorrectly aligned. BAQ is not a traditional recalibration algorithm in the sense that it does not attempt to adjust quality scores so that they better reflect the probability of a sequencing error. To account for incorrectly aligned bases, original quality scores are adjusted by taking the minimum of the original quality score and the BAQ score. BAQ has been shown to improve the accuracy of SNP discovery and resolve false SNPs caused by indels [7]. There are four main differences between the methods of ReQON and BAQ.

1. BAQ is implemented in the SAMtools software package [9] which runs on Unix. ReQON is an R package.

2. BAQ requires a reference sequence file for the entire genome. ReQON only requires reference sequence for the specified training region.

3. BAQ can only decrease the base quality score. ReQON allows quality scores to be adjusted both higher and lower.

4. BAQ only adjusts quality scores to account for alignment errors. We believe that alignment errors and uncertainty should be reflected in the mapping quality score and that the base quality score should correspond to the probability that the base is a sequencing error. To separate the effects of sequencing errors from alignment errors, ReQON filters out bases that may be alignment errors from the training set before training the model.

In order to assess the accuracy of all three methods, Table 1 compares the FWSE statistic between ReQON, GATK and BAQ for two separate chromosomes, 10 and 20, on the two cell line replicates. Note that while GATK and BAQ train their models on the entire genome, ReQON only trained its model on chromosome 10. In this regard, chromosome 20 can be viewed as an independent testing set when assessing model performance for ReQON but not GATK or BAQ. Error calls were made in the same manner as the ReQON algorithm, which we feel more accurately identifies true sequencing errors, as described previously. In every case, FWSE is much lower for quality scores recalibrated with ReQON than GATK or BAQ. From this, we can conclude that the recalibrated quality scores from ReQON more accurately represent the probability that a base is a sequencing error.

In order to determine how well GATK and BAQ quality scores distinguish between non-reference bases classified as sequencing errors and non-reference bases belonging to positions of known variants, the discrimination analysis was repeated using the GATK and BAQ recalibrated quality scores. Table 2 shows the AUC for the original quality scores and the recalibrated quality scores. All of the recalibration methods outperform the original quality scores for both cell line replicates. The discrimination performance of GATK and ReQON is approximately the same, with GATK performing better in one replicate and ReQON performing better in the other replicate, and both outperforming BAQ.

## Discussion

The previous analyses show that the original quality scores assigned by the sequencer machine are neither accurate nor do a good job of discriminating sequencing errors from non-errors. ReQON, GATK and BAQ produce much more reliable quality scores, although the quality scores assigned by ReQON are more accurate than those assigned by GATK or BAQ (Table 1). In most cases, any of the three recalibration algorithms is reasonable to use, but there are some distinct differences.

The algorithms of ReQON and GATK consider very similar covariates, yet Tables 1 and 2 show that ReQON performs just as well as, and in most cases better than, GATK. ReQON’s better performance than GATK can be attributed to two main differences: recalibrating low-quality bases and filtering out mismatch bases that may be due to true variants or mapping errors.

First, as previously mentioned, GATK chooses not to recalibrate bases with very low quality scores, with the default quality threshold set at 5. Their reasoning is that these quality scores indicate bad or randomly called bases by the sequencer, so these original qualities should be kept as is. This makes sense if these low-quality bases are filtered out before later analyses. However, investigators may prefer not to filter out low-quality bases, such as when sequencing experiments are expected to yield low coverage. Under this low coverage setting, it makes more sense to recalibrate all bases, regardless of quality score, which is how ReQON operates. Due to this difference, in general, the low quality bases of GATK have poor accuracy because they are not recalibrated. In contrast, the recalibrated low-quality scores from ReQON are much more accurate.

The low-quality bases that GATK chooses not to recalibrate have a large contribution to its FWSE (Table 1), indicating decreased accuracy. If these low qualities are removed from the analysis, then FWSE is approximately equal between GATK and ReQON. For a more exact comparison, we could have changed the threshold for GATK and recalibrated all bases regardless of its original quality score. But, as most users will use the default settings when running either recalibration algorithm, we choose to only compare the output using these default settings.

A second main advantage of ReQON over GATK is the criterion used for identifying sequencing errors. GATK identifies sequencing errors by filtering out known variant positions, then calling all bases that do not match the reference sequence as errors. In reality, some of these bases will not be sequencing errors but will be correct calls, such as novel variants or mapping errors. These miscalls disproportionately affect the higher quality scores. Because GATK's observed error rate is approximately the sum of the sequencing error rate, the alignment error rate and the rate of novel variants, GATK will be underestimating the true quality. In contrast, ReQON goes a step further by utilizing information from multiple reads and removing positions from the training set with low confidence in the error calls (determined by model parameters nerr and nraf). Figure 3 shows an example of such a position. For cell line replicate 1, ReQON removed 77,133 bases at 2,117 positions (average of 36x coverage) from the training set that GATK called as sequencing errors. These removed positions are likely to be novel variants or mismatches due to systematic alignment errors. ReQON identifies these positions without prior knowledge; in contrast, GATK would need information about these positions in order to remove them when building its model. Therefore, ReQON should be preferred when the data are aligned to unfinished genomes, where many mapping errors are expected, or when an input file of known variant positions is not available.

Figure 3 also shows that ReQON assigns significantly higher quality scores to the non-reference bases at this position than GATK (two-sided paired *t*-test, p = 8.36 × 10^-9^). This suggests that GATK biases against discovering novel variants by assigning lower quality scores to non-reference bases at positions supported by multiple reads. Therefore, investigators interested in detecting novel variants should prefer ReQON over GATK.

An additional main difference between the recalibration algorithms is that GATK tests model performance on the same data that were used in training the model (the entire genome). This approach leads to over fitting and overly optimistic estimates of the error. This issue, along with the concern of falsely identifying non-reference bases as sequencing errors, calls into question the true performance of GATK recalibration. On GATK’s software website [15], the authors discuss the option of training their model on a smaller subset of the data to reduce runtime. The authors provide evidence that training the model on a subset of the data leads to decreased accuracy. They conclude that users interested in maximum recalibration accuracy should continue to train on the full data set. We view this as further evidence that the GATK model overfits to the training data and, thus, overestimates the true model performance. In contrast, ReQON trains the model on a training set, which allows performance to be measured on a separate testing set. The analyses presented in the Results section show that ReQON does not overfit the model to the training data.

ReQON was also compared to BAQ, which is not a traditional quality score recalibration algorithm in the sense that it does not attempt to adjust quality scores so that they better reflect the probability of a sequencing error. Instead, BAQ only considers alignment quality and adjusts the base quality when the alignment quality is low. Due to the difference in motivation, ReQON greatly outperforms BAQ in terms of accurately representing the probability of a sequencing error, shown in Table 1. BAQ adjustment has been shown to improve SNP calling [7], especially in reducing false calls at positions near indels. Table 2 shows that ReQON does a better job at distinguishing non-reference bases belonging to dbSNP, representing true variants, from non-reference bases at other positions, representing mainly sequencing errors with possibly a few novel variants. Although more detailed analysis is required, this suggests that, overall, ReQON may be as effective at improving variant calling as BAQ.

Like all available base quality score recalibration algorithms, the ReQON results will be dependent on the accuracy of the read alignments. Alignment becomes much more complicated when considering indels, variants, splicing or poorly annotated genomes. BAQ incorporates mapping quality into its recalibrated scores. However, as seen in Table 1, this comes at the cost of the quality scores accurately representing the probability that a base is a sequencing error. We believe that alignment quality should be represented in mapping quality scores and that base quality scores should only convey information about the likelihood of a base being a sequencing error. ReQON attempts to separate out mismatches due to alignment by identifying and removing such bases from the training set. Following the assumption that mapping errors occur in a more systematic fashion than stochastic sequencing errors, this filtering is achieved through the use of parameters nraf and nerr. While this may not remove all effects of alignment on the quality score, it demonstrates a marked improvement over GATK which fails to consider alignment-specific sources of error.

## Conclusions

The results presented here demonstrate the need for quality score recalibration, especially if these quality scores are used in downstream analyses. We presented a novel R package, ReQON, which produces quality scores that are both more accurate, in the sense that they more closely correspond to the probability of a sequencing error, and do a better job of discriminating between sequencing errors and non-errors. ReQON was compared to two of the most commonly used recalibration algorithms, GATK and BAQ. All recalibration algorithms significantly outperform the original quality scores in terms of accuracy and discrimination performance. ReQON produces more accurate quality scores than GATK and BAQ, although in most cases, any recalibration algorithm is reasonable to use. However, due to differences in the underlying assumptions and model used to recalibrate the quality scores, we strongly recommend recalibrating with ReQON when trying to identify novel variants, or when aligning to genomes that are unfinished or have incomplete databases of known variant positions.

## Methods

Two replicates of RNA from the U87 glioblastoma cell line [11] were sequenced using Illumina’s Genome Analyzer II, representing identical sequence runs but of slightly differing quality. Each run produced 76 base pair single-end reads which were aligned to the human genome reference version 19 (hg19) using Map Splice [16]. The aligned reads were sorted and indexed using SAMtools [9].

For both cell line replicates, ReQON was run using the default settings. Region was set to chromosome 10 and input for RefSeq was hg19 and SNP was dbSNP version 132 [12].

The same two cell line replicates were also recalibrated using GATK [3] and the BAQ option in SAM tools [7] for comparison. Similar to ReQON, hg19 was used as the reference sequence. GATK recalibration used dbSNP version 132 as the variant file and the following covariates: ReadGroupCovariate, QualityScoreCovariate, DinucCovariate and CycleCovariate. BAQ quality adjustment was performed using SAMtools *calmd* function with options –AEr.

## Availability and requirements

· **Project name:** ReQON (version 1.3.8 or higher).

· **Project homepage:**http://bioconductor.org/packages/devel/bioc/html/ReQON.html.

· **Operating system(s):** Platform independent.

· **Programming language:** R (version 2.15 or higher).

· **Other requirements:** Bioconductor, Java 1.6 or higher.

· **License:** GPL version 2.

· **Any restrictions to use by non-academics:** none.

## Competing interests

The authors declare that they have no competing interests.

## Authors’ contributions

CRC, KCW, CMP, JSM and DNH formulated the problem and developed the method. CRC, KC and CB wrote the software. CRC, MDW and JSP performed the data analysis. CRC, JSM and DNH wrote the manuscript. All authors read and approved the final manuscript.
